# Sustained release of VEGF from PLGA nanoparticles embedded thermo-sensitive hydrogel in full-thickness porcine bladder acellular matrix

**DOI:** 10.1186/1556-276X-6-312

**Published:** 2011-04-07

**Authors:** Hongquan Geng, Hua Song, Jun Qi, Daxiang Cui

**Affiliations:** 1 Department of Pediatric Urology, Xinhua Hospital, Shanghai Jiao Tong University School of Medicine, Shanghai 200092, People's Republic of China; 2 Department of Bio-Nano Science and Engineering, National Key Laboratory of Nano/Micro Fabrication Technology, Key Laboratory for Thin Film and Microfabrication of Ministry of Education, Institute of Micro-Nano Science and Technology, Shanghai Jiao Tong University, 800 Dongchuan Road, Shanghai 200240, People's Republic of China; 3 Department of Urology, Xinhua Hospital, Shanghai Jiao Tong University School of Medicine, Shanghai200092, People's Republic of China

## Abstract

We fabricated a novel vascular endothelial growth factor (VEGF)-loaded poly(lactic-*co*-glycolic acid) (PLGA)-nanoparticles (NPs)-embedded thermo-sensitive hydrogel in porcine bladder acellular matrix allograft (BAMA) system, which is designed for achieving a sustained release of VEGF protein, and embedding the protein carrier into the BAMA. We identified and optimized various formulations and process parameters to get the preferred particle size, entrapment, and polydispersibility of the VEGF-NPs, and incorporated the VEGF-NPs into the (poly(ethylene oxide)-poly(propylene oxide)-poly(ethylene oxide) (Pluronic^®^) F127 to achieve the preferred VEGF-NPs thermo-sensitive gel system. Then the thermal behavior of the system was proven by *in vitro *and *in vivo *study, and the kinetic-sustained release profile of the system embedded in porcine bladder acellular matrix was investigated. Results indicated that the bioactivity of the encapsulated VEGF released from the NPs was reserved, and the VEGF-NPs thermo-sensitive gel system can achieve sol-gel transmission successfully at appropriate temperature. Furthermore, the system can create a satisfactory tissue-compatible environment and an effective VEGF-sustained release approach. In conclusion, a novel VEGF-loaded PLGA NPs-embedded thermo-sensitive hydrogel in porcine BAMA system is successfully prepared, to provide a promising way for deficient bladder reconstruction therapy.

## Introduction

A variety of congenital and acquired conditions cause compromised bladder capacity and compliance. The major surgical solution is enterocystoplasty, whereby the functionally deficient bladder is reconstructed using biomaterials. In terms of biomaterials for bladder reconstruction, bladder acellular matrix allograft (BAMA) [1,2] has great potential for complete and functional regeneration of the bladder. BAMA is a naturally derived biodegradable material that is currently being developed for use as a bladder substitute. It is produced by extracting the cells and soluble matrix components from the extracellular matrix, and so it has almost all the properties of a normal bladder, and maintains a low potential for inflammatory attack on the graft because most of the antigenic proteins are extracted from the bladder tissue. The long-term follow-up of vascular acellular matrix allografts has demonstrated their biocompatibility [3-5].

Previous research has proven that the administration of growth factors can promote tissue revascularization. Under an appropriate dosage, pro-angiogenic cytokines, such as vascular endothelial growth factor (VEGF) [6,7], can up-regulate angiogenesis by signaling vascular endothelial cells to undergo proliferation, migration, and differentiation into new blood vessels. However, the short-lived effect and high instability (such as oxidation, deamidation, and diketopiperazine formation in a physiological environment) of the VEGF protein result in some disappointing clinical trials, because the therapeutic effects of the protein can only be achieved at extremely high doses, which often results in side effects such as the progression of malignant vascular tumors [8]. A superior formulation is needed to deliver VEGF continuously to maintain the VEGF concentration within the therapeutic window during the long term of tissues' reconstruction.

In this study, we report a novel VEGF-loaded nanoparticles (NPs)-embedded porcine bladder acellular matrix with thermo-response system, which is designed for achieving a sustained release of VEGF protein, and embedding the protein carrier into the BAMA. For the incorporation and sustained release of VEGF, the protein was encapsulated in NPs with biodegradable poly(lactic-*co*-glycolic acid) (PLGA) by multi-emulsion and solvent evaporation methods, which result in the protein-loaded round-shaped NPs [9,10]. Then, the VEGF-loaded PLGA NPs are combined with a hydrophilic gel matrix, (poly(ethylene oxide)-poly(propylene oxide)-poly(ethylene oxide) (Pluronic^®^) F127 hydrogel [11], using the sol-gel transition to give a well-dispersed PLGA particles-embedded hydrogel [12]. Finally, the VEGF-NPs-F127 gel was embedded in BAMA with multipoint injection. Such a strategy as this allows the carrier system to show a sustained release of protein, a retention of protein-loaded NPs in BAMA, as well as additional properties such as thermo-sensitivity and biocompatibility.

## Experimental

### Materials

Pluronic^® ^F127 triblock copolymer, Tween^® ^80 (polyoxyethylene sorbitan monooleate), and poly(vinyl alcohol) (PVA) (Mw 14-16 kDa) were purchased from Sigma-Aldrich (Shanghai, China). PLGA with a monomer ratio (lactic acid/glycolic acid) of 50:50 was purchased from Daigang Biomaterial Co., Ltd. (Jinan, China). rhVEGF_165 _and rhVEGF enzyme-linked immunosorbent assay (ELISA) kit was purchased from Peprotech (Rocky Hill, NJ, USA), and all other reagents were purchased from Sigma-Aldrich.

### Preparation of NPs-embedded thermo-sensitive hydrogel

PLGA NPs containing VEGF (0.1 μg/mg of NPs) were prepared by the double emulsion-solvent evaporation technique based on the method of Liao et al. [13]. In briefly, 20 mg of PLGA was dissolved in appropriate amount of dichloromethane. This polymer solution was injected into 100 μL of phosphate-buffered saline, pH7.4 (PBS7.4) as the inner aqueous phase (W1) containing VEGF, heparin (Hp, 16 kDa), and human serum albumin (HSA) (VEGF/Hp/HSA 1:1:500, w/w/w). Next, the previously formed inner emulsion (W1/O) was generated by a high-speed homogenizer of IKA ultra turrax operating at 3,000 rpm for 2 min. Then, the first emulsion was injected into 10 mL outer aqueous phase (W2), which was composed of aqueous 1.5% (w/v) PVA and 2% Tween80, resulting in a multiple emulsion (W1/O/W2), which was homogenized by ultra turrax at specific speed and time following an incubation on ice. This emulsion was put on a rota-evaporator under vacuum (500 mHg) for 3 h at room temperature for complete solvent evaporation. The organic phase was evaporated leading to precipitation of polymer to get the NPs, which hardens over time. The NPs were collected by centrifugation at 10,000 × *g *for 5 min at 4°C and washed with distilled water three times followed by freeze-drying using mannitol as cryoprotectant (PLGA:Mannitol:100:30) to get dry powder containing NPs.

In this process, several factors impact the formation of NPs with acceptable size, polydispersity, and good entrapment efficiency. Based on preliminary studies, under the premise of specific PLGA and external aqueous phase stabilizer, three critical factors, namely, volume ratio of organic solvent phase to external aqueous phase, agitation speed, and duration of homogenization were selected for the optimization of mean particle size and entrapment efficiency. During the optimization trials, these values for critical factors were varied between the extreme levels. In the present design, 15 different experiments were carried out to identify the optimum level of the major variables as indicated in Table 1.

**Table 1:** Experimental design matrix with observed values of the objectives variables of protein-loaded NPs

Experiment Code	X1	X2	X3	Z-Ave^a ^(nm)	Polydispersibility Indice	E.E.(%) ± SD (*n *= 6)
1	8.2	5	0.66	280.8	0.483	35.29 ± 0.07
2	8.2	5	0.24	309.5	0.286	38.82 ± 0.53
3	8.2	2	0.66	602.6	0.702	51.76 ± 0.28
4	8.2	2	0.24	1428.7	0.632	50.59 ± 0.46
5	5.5	6	0.45	291.2	0.378	48.82 ± 0.12
6	5.5	1	0.45	890.7	0.806	54.71 ± 0.09
7	5.5	3.5	0.8	355.4	0.567	42.94 ± 0.14
8	5.5	3.5	0.1	640.2	0.686	55.88 ± 0.10
9	2.8	5	0.66	337.0	0.492	44.12 ± 0.74
10	2.8	5	0.24	345.8	0.384	45.88 ± 0.67
11	2.8	2	0.66	651.0	0.712	50.00 ± 0.41
12	2.8	2	0.24	2503.5	0.762	52.35 ± 0.75
13	10	3.5	0.45	380.5	0.536	44.71 ± 0.69
14	1	3.5	0.45	1025.5	0.691	49.41 ± 0.21
15	5.5	3.5	0.45	429.2	0.555	47.06 ± 0.55

X1 represents duration of homogenization (min); X2 represents agitation speed (krpm); X3 represents volume ratio of organic solvent phase to external aqueous phase (V/V); Z-Ave represents average particle size diameter; E.E. represents encapsulation efficiency.

Fluorescent probes-loaded NPs were obtained by adding hydrophilic CdTe quantum dots (QDs) (1 μg/mg of NPs, prepared according to our previous report with maximum emission wavelength of 590 nm) [14] into the inner aqueous phase instead of VEGF protein, and the NPs prepared as described produced optimized results. The NPs containing only Hp and HSA were produced as negative control.

Then, the accurate NPs (lyophilized) were resuspended in distilled water. This suspension was added to the concentrated F127 solution so that the final F127 concentration reached 25% w/v and stirred gently for 10 min after incubation on ice for uniform distribution of NPs in the F127 solution.

### NPs morphology and particle size

The formulations prepared by double emulsification solvent evaporation were performed for shape and surface morphology using a Zeiss Ultra 55 scanning electron microscope (SEM). The dried NP samples were suspended in distilled water until further examination.

Particle diameter was determined using a Nicomp 380ZLS particle sizing system. Accordingly, the dried NP samples were suspended in distilled water. The obtained homogenous suspensions were examined to determine the mean diameter and polydispersity index.

### Determination of encapsulation efficiency

The NP encapsulation efficiency (E.E.) was determined upon their separation from the aqueous preparation medium containing the non-associated protein by centrifugation (20,000 × *g*, 4°C, 10 min). The amount of free protein was determined in the supernatant using a bicinchoninic acid assay. The extraction procedure was performed for a total of 3 × for each particle type. The NP E.E. was calculated using the following equation: E.E. (%) = [(Total protein amount - Free protein amount)/Total protein amount] × 100%.

### Gelation temperature and thermo-reversible behavior *in vivo*

The gelation temperatures of varying concentration (w/v) of Pluronic F127 were determined by the tube inversion method. In brief, accurate F127 was dissolved in ultrapure water at cold temperature in Eppendorf tube, the tubes were reversed constantly and the temperature at which the solution stopped dropping was measured; at this temperature, the solution was converted into gel. The thermo-reversibility was verified by giving repeated cooling and heating cycles to confirm any change in the gelation temperature and reversibility of gel-sol behavior.

To confirm the thermo-sensitive, sustained release properties of the NPs-embedded hydrogel, physiologically normal nude mice were treated with QDs-NPs-F127 gel, QDs-NPs, and QDs-physiological saline solution, and they were all treated with aliquots QDs dosage of 2 mg/kg via subcutaneous injection. *In vivo *mouse images were acquired using a Berthold Night OWL *in vivo *imager. Fluorescence images of all the experimental mice were taken continuously for 24 h along with the typical images at 10 min post-injection.

### Release kinetics

*In vitro *drug release of VEGF-loaded NPs-embedded thermo-sensitive hydrogel was evaluated in buffer solution. In brief, 10 mg of dried NPs was suspended in 0.2 mL 25% Pluronic^® ^F127 solution, and then the solution was rapidly pushed into a 2 cm^2 ^full-thickness porcine bladder acellular matrix through multipoint sequential injection. The matrix was dipped into 2 mL phosphate buffer saline (PBS) at pH 7.4 and at 37°C, which had previously been filtered on 0.22-μm sterile filters and microbiologically preserved with 0.02%w/w sodium azide. Then, the release media were placed in a thermostatic bath at 37°C. At scheduled time intervals, the release medium was withdrawn and replaced with the equal volume of fresh, filtered medium. Release of VEGF from NPs, 25% Pluronic^® ^F127, and NPs-embedded 25% Pluronic^® ^F127 were tested at the same time as references. Samples were centrifuged (20,000 × *g*, 4°C, 10 min), and the supernatant was analyzed for VEGF content via ELISA using the VEGF ELISA kit as per the manufacturer's protocol. Results are expressed as cumulative release of VEGF NPs ± SD (standard deviation) of three replicates.

### Bioactivity of released VEGF

The bioactivity of the VEGF released from the microparticles was evaluated *in vitro *by determining the proliferative capacity of an endothelial cell line (human umbilical vein endothelial cell, HUVEC) after VEGF treatment. First, VEGF-loaded NPs were incubated in Endothelial Cell Growth Medium-2 (EGM-2) without growth factors for a continuous period of 1, 5, 10, or 15 days, the release EGM-2 medium was filtered with 0.22-μm sterile filters and VEGF values were measured using ELISA and stored at 4°C. Second, HUVEC were cultured in EGM-2 media supplemented with 30 μg/mL endothelial cell growth supplement, 10% fetal bovine serum, 1% Hp, and 1% penicillin/streptomycin. In order to determine the endothelial cell proliferation capacity after VEGF stimulation, the HUVEC were placed into 96-well culture plates with a density of 1 × 10^3^cells/well and allowed to adhere overnight. Medium was then aspirated, and the released EGM-2 medium supernatant from VEGF-loaded NPs was then added to wells immediately to make an equivalent final VEGF concentration of 10 or 20 ng/mL. Native, non-encapsulated VEGF at 10 or 20 ng/mL was used as the 100% bioactivity benchmark, and wells with medium only (no VEGF), as well as the released EGM-2 medium supernatant from non-loaded NPs, were employed as the negative control. Cells were incubated for 3 days, and the number of viable cells in each experimental group was determined by the 3-(4,5-dimethylthiazol-2-yl)-2,5-diphenyltetrazolium bromide assay.

### Data presentation and statistical analyses

Unless otherwise indicated, data are represented as mean ± SD. Statistical significance was determined using a student's *t*-test with a 95% confidence interval, unless otherwise noted. Statistical calculations were performed using a SPSS software.

## Results and discussion

Preparation of NPs is a complex procedure as it involves several processing variables and design components [15,16]. Even slight changes of these variables and system components can have significant impact on the quality of final product. Unique attributes of NPs such as particle size and entrapment efficiency are of utmost importance from the biological and pharmaceutical point of view [17-19]. Particles with smaller average diameter showed slower release. Smaller particles are generally formed with higher impact [20]. It varies the tortuous polymeric diffusion pathways in smaller particles [21]. This ultimately leads to a sustained diffusion of protein from the particles. Another quality attribute of NPs is the entrapment efficiency which should be properly optimized to avoid the loss of drug during processing [22]. It is of the highest importance especially in case of drugs such as VEGF, with their cost being very high. Here, we choose the particle diameter and the entrapment efficiency as the experimental investigation response. Using the double emulsion method for preparation of NPs, the second emulsification is decisive for the size of the NPs. Thus, the intensity and the time of emulsification can be used for controlling size. Here, we selected three critical factors, namely, volume ratio of organic solvent phase to external aqueous phase, agitation speed, and duration of homogenization for the optimization of the experimental investigation response.

Table 1 depicts the various process parameters of the prepared protein containing NPs. The results of the particle size analysis by laser diffraction showed that particle sizes varied from 280.8 to 2503.5 nm with variable polydispersity indices among the experimental formulations. Among them, formulation 1 had the least value of the average diameter, whereas 12 had the maximum. The polydispersibility indices also showed the similar patterns of dispersibilities, i.e., formulation 2 had the least value and 6 had the maximum. The data suggest that with increasing homogenizing speed, average diameters of particles were reduced. However, when speed of homogenization was further enhanced, the protein E.E. reduced too, Therefore, after all these results having been considered, experiment 5 was concluded to be the optimized one for the preparation of the protein-loaded NPs.

**Figure 1:**
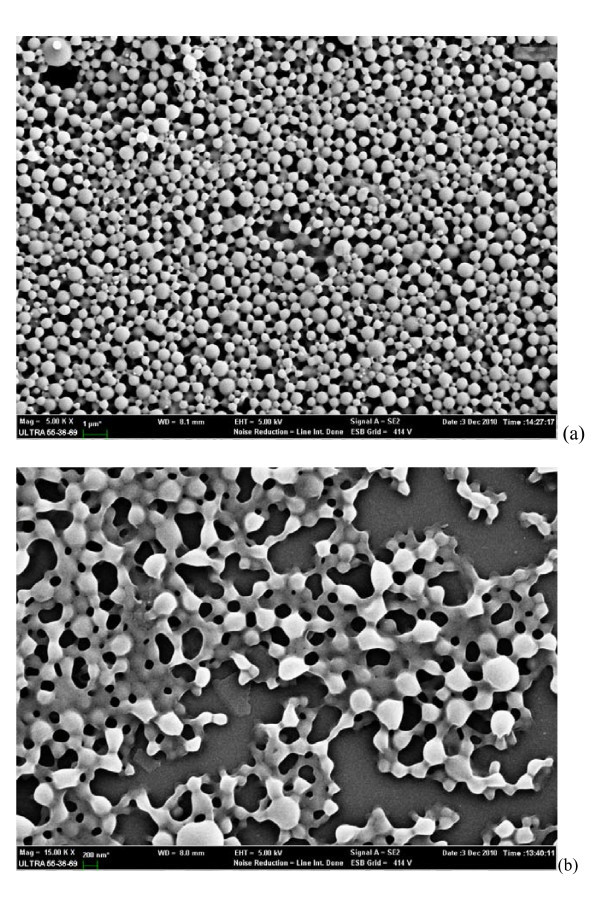
**SEM images of NPs**: **(a) **free NPs, and **(b) **NPs embedded in Pluronic F127 gel.

The SEM study (Figure 1a) shows smooth, homogeneous, and spherical-shaped images in nano range, and there is no aggregation after lyophilization in case of experiment formulation code 5. Approximately more than 90% particles were found to have diameter below 600 nm. The average particle size was about 300 nm, and the densest and the narrowest range of particle dispersion was noticed between 100 and 400 nm.

**Figure 2:**
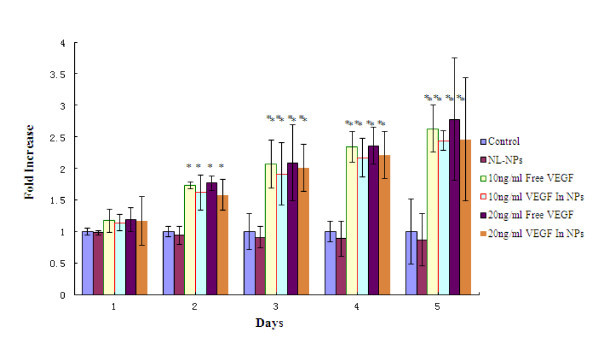
**Proliferation of HUVEC cells was induced by 10 ng/mL free-VEGF, or 20 ng/mL free-VEGF, or 10 ng/mL VEGF in NPs, or 20 ng/mL VEGF in NPs, or non-loaded NPs (NL-NPs) at the same concentration of PLGA with the application described above, and compared to culture medium alone (control) for 1-5 days**. *Y*-axis represents fold increase versus control. Asterisk represents *P *< 0.05 and double asterisk represents *P *< 0.01.

The bioactivity of the encapsulated VEGF released from the NPs was examined by determining its capacity to induce proliferation of endothelial cells (HUVEC) (Figure 2). VEGF-NPs (10 or 20 ng/mL) induced a 2-2.5-fold increase in proliferation of HUVEC in comparison with control (no VEGF) or non-loaded NPs (NL-NPs) after 3 days in culture (*P *< 0.01). This increase was similar to that observed when HUVEC cells were cultured with addition of free-VEGF at doses of 10 or 20 ng/mL. The results show NL-NPs caused little reduction in cell viability compared with the control, but there was not any significant statistical difference between them, indicating that NL-NPs were better tolerated at the experiment's concentration. Furthermore, similar levels of stimulation in the HUVEC cells treated either with the free-VEGF or the VEGF-NPs were detected, confirming that the process of encapsulation does not affect negatively VEGF biological activity significantly.

**Figure 3:**
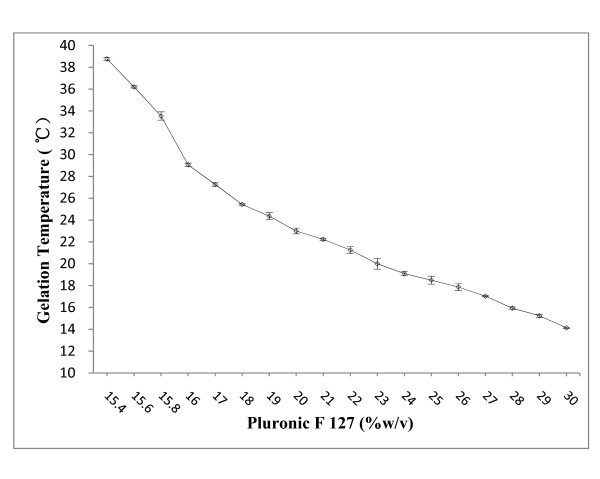
**Gelation temperature and thermoreversible behavior of Pluronic F 127 gel**.

**Figure 4:**
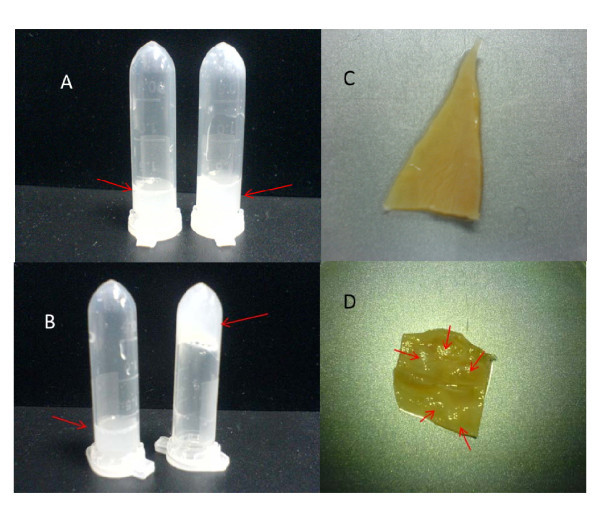
**Gel-to-sol transition behavior of NPs-F127 solution**. **(a) **Solution state of 50 mg/mL NPs in 20% F127 (left) and in 25% F127 (right) at 15°C; **(b) **solution state of 50 mg/mL NPs in 20% F127 (left) and in 25% F127 (right) at 20°C; **(c) **an porcine bladder acellular matrix; and **(d) **an porcine bladder acellular matrix been treated with VEGF-NPs-F127 gel by multipoint sequential injection.

It is noteworthy to mention again that an appropriate sol-gel temperature, gelation, and maintaining of its consistency after injection of the block copolymer solution, were crucial for its utilization for various applications. Tube inversion has been used previously by several groups to determine the gel boundary of gel-sol behavior [23]. Thermoreversible sol-gel transition of F127 aqueous solution originates from micelle formation and micelle volume change owing to PEO/water, and PPO/water's lower critical solution temperature (LCST) behavior [24]. Above LCST temperature of PPO, the micelle with PPO core and PEO shell appears. As temperature increases, the number of micelles increases. At high temperature, interaction of PEO and water is unfavorable, and therefore, gel-to-sol transition occurs because of dehydration and shrinking of PEO shell. Above PEO-water LCST temperature, phase separation between polymer and water is observed. As illustrated in Figure 3, gelation temperature decreased with increase of the concentration of F127 and decreased proportionally to the concentration. Solutions containing less than 15.4% F127 did not form gels over the tested temperature range, while a F127 concentration higher than 30% led to difficulty in preparation and administration. In this study, approximately 25% of F127 was required to obtain NPs hydrogel formulation with the transition temperature of approx. 20°C (Figure 4a, b).

**Figure 5:**
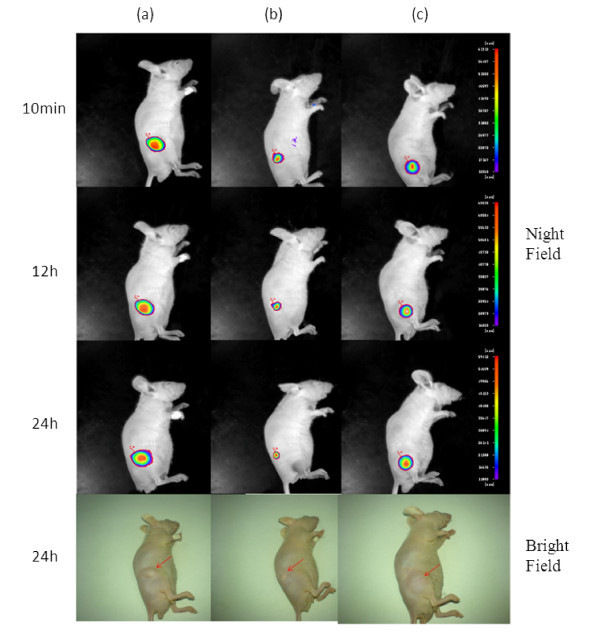
***In vivo *thermal behavior fluorescence imaging test using physiologically normal nude mice**: **(a) **nude mouse treated with QDs-NPs-F127 gel, **(b) **nude mouse treated with QDs-NPs, and **(c) **nude mouse treated with QDs-physiological saline solution. All mice were treated with aliquots QDs dosage of 2 mg/kg via subcutaneous injection. Intensity bar shows the fluorescence intensity level.

Figure 5 exhibits the typical fluorescence images of different healthy group mice from 10-min to 24-h post-injection. Figure 5a represents the image of the mice with QDs-NPs-F127 gel treatment on the right leg, and bright fluorescence signal was observed at10 min; after 24 h, the intensity of the fluorescence signal did not vanish. Obviously, this may possibly be due to the rapid sol-gel transmission of the QDs-NPs-F127 gel. Furthermore, the shape of the injectant below the skin of the mouse was maintained in smooth and clear condition after 24 h, and the tissues around the injectant did not induce any inflammatory reaction, representing the superior biocompatibility of the QDs-NPs-F127 gel. Figure 5b represents the fluorescence images of the mice with QDs-NPs treatment; the images show that the fluorescence signal was aggregated and weakened rapidly after injection, the solvent of the injectant was rapidly absorbed simultaneously, and the NPs were compressed and degraded in accelerated manner. Figure 5c represents the images of the mice with QDs-physiological saline solution, while a curious inflammatory reaction, the swelling, and distension at the administration site were observed after 24 h of post-injection, and this may be attributed to the serious toxicity of the CdTe QDs.

**Figure 6:**
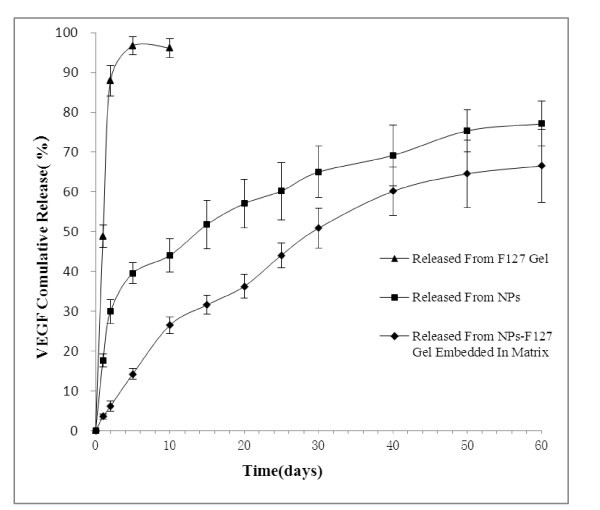
***In vitro *cumulative release of VEGF from PLGA NPs in PBS at pH 7.4 and 37°C**.

The *in vitro *release kinetics was performed in PBS (pH 7.4) at 37°C for 60 days as reported in Figure 6. In this study, VEGF released from NPs within the first 2 days (burst effect) was 30 ± 3%, followed by a phase of sustained release with almost 75% of VEGF being released within 60 days. The VEGF release from NPs-F127 gel embedded in full-thickness acellular porcine bladder matrix (Figure 4c, d) was slower than that from VEGF-NPs (almost 60% of VEGF being released within 60 days). The burst effect was decreased below 15 ± 2%, which might be due to longer diffusion pathways of VEGF in porcine bladder acellular matrix. In addition, sustained release of VEGF from simple F127 gel was not remarkable compared with the two groups described above.

## Conclusion

A thermo-sensitive hydrogel-entrapped VEGF-NPs system has been prepared and characterized in this study. Various formulations and process parameters were identified and optimized to obtain the preferred particle size, entrapment, and polydispersibility of the VEGF-NPs system. Then, the thermo-sensitive behavior was proven by the *in vitro *and *in vivo *study, and the kinetic sustained release profile of the VEGF-NPs-F127 gel system embedded in porcine bladder acellular matrix was investigated. Results indicate that the thermal responsive VEGF-NPs-F127 gel system prevents acute tissue reaction, inflammation, and toxic manifestation because the gel creates a tissue-compatible environment and an effective VEGF sustained release approach. The proposed system provides a promising way for deficient bladder reconstruction therapy. Entrapment of growth factor drugs into this kind of nanohydrogels for deficient bladder reconstruction therapy will form the scope of our future study.

## Abbreviations

BAMA: bladder acellular matrix allograft; ELISA: enzyme-linked immunosorbent assay; E.E.: encapsulation efficiency; HSA: human serum albumin; HUVEC: human umbilical vein endothelial cell; NPs: nanoparticles; PLGA: poly(lactic-*co*-glycolic acid); PVA: poly(vinyl alcohol); QDs: quantum dots; SEM: scanning electron microscope; PBS: phosphate buffer saline; VEGF: vascular endothelial growth factor.

## Competing interests

In the past five years, all the authors haven't received any reimbursements, fees, funding, or salary from an organization that may in any way gain or lose financially from the publication of this manuscript, either now or in the future.

All the authors of this paper haven't hold any stocks or shares in any organizations that may in any way gain or lose financially from the publication of this manuscript.

All the authors of this paper haven't hold or applied any patents relating to the content of the manuscript, and all the authors haven't received reimbursements, fees, funding, or salary from any organizations that hold or have applied for patents relating to the content of the manuscript.

All the authors of this paper haven't any non-financial competing interests (political, personal, religious, ideological, academic, intellectual, commercial or any other) to declare in relation to this manuscript.

In conclusion, all the authors declare that no competing interests exist in this paper.

## Authors' contributions section

Hongquan Geng and Hua Song prepared the NPs-embedded thermo-sensitive hydrogel. Hongquan Geng characterized NPs and determined the encapsulation effciency. Hua Song studied the *in vitro *drug release, fluorescence image of the mice with QDs-NPs-F127 gel, determined the bioactivity of released VEGF and drafted the manuscript. Jun Qi and Daxiang Cui conceived of the study, and participated in its design and revised the manuscript.
